# The mediating effect of trust in the relationship between transformational leadership of university sports coach and team task cohesion

**DOI:** 10.3389/fpsyg.2026.1736310

**Published:** 2026-03-10

**Authors:** Sujin Kim, Inwoo Kim

**Affiliations:** Department of Sports Science Convergence, Dongguk University, Seoul, Republic of Korea

**Keywords:** affective trust, cognitive trust, team task cohesion, transformational leadership, university sport

## Abstract

**Introduction:**

Transformational leadership is crucial for team functioning and cohesion in sports, yet the psychological mechanisms, specifically the role of trust, linking this leadership style to team outcomes in competitive university contexts remain under-investigated. This study, therefore, examined the associations and potential mediating roles of cognitive and affective trust in the relationship between athletes’ perceptions of transformational leadership and team task cohesion.

**Methods:**

Data were collected from 362 male university soccer players affiliated with the Korea University Sports Federation (KUSF) using stratified sampling and standardized self-report instruments. To examine the hypothesized relationships, we employed structural equation modeling (SEM), focusing on the direct and indirect effects of cognitive and affective trust as potential mediators.

**Results:**

The analysis indicated that transformational leadership had no significant direct association with team task cohesion. However, cognitive trust was found to function as the sole significant mediator in this relationship. Although affective trust was positively associated with transformational leadership and cognitive trust, it did not significantly relate to task cohesion. Furthermore, cognitive trust significantly predicted affective trust, consistent with a unidirectional sequence from rational appraisal to emotional bonding.

**Discussion:**

These findings offer empirical support for the pivotal role of cognitive trust as a foundational mediator that simultaneously facilitates performance-oriented outcomes and interpersonal attachment in university sports. Specifically, the results extend theoretical understanding by clarifying that cognitive trust serves as a critical gateway, linking transformational leadership to both greater team task cohesion and the progressive development of affective trust among athletes. Practically, the results emphasize that coaches must prioritize consistent, competent, and fair leadership behaviors to cultivate cognitive trust; this strategic focus activates a dual psychological pathway that effectively enhances task-oriented cohesion while also strengthening the long-term emotional stability of the team.

## 1 Introduction

In organizational settings, leaders play a pivotal role in establishing direction, motivating members, and managing resources effectively to achieve overarching goals (Ji and Chah, 2020). Within the disciplines of organizational behavior and management, leadership has long been regarded as a core determinant of organizational success and sustainability. Among various leadership paradigms, transformational leadership has drawn particular attention for its demonstrated ability to inspire intrinsic motivation and articulate a clear sense of purpose and vision, thereby enhancing overall organizational performance (Bass and Avolio, 1994).

Transformational leadership is defined by a leader’s capacity to help followers recognize their potential and inspire them to achieve outcomes that exceed expectations. It encompasses four fundamental dimensions: idealized influence, inspirational motivation, individualized consideration, and intellectual stimulation (Bass, 1985; Bass, 1985). Leaders who embody this style uphold strong ethical principles and serve as role models while nurturing followers’ personal growth, creativity, and autonomy (Bass and Riggio, 2006). By articulating a compelling organizational vision and communicating it effectively, transformational leaders cultivate an environment in which individuals internalize the meaning of their work and engage with a sense of autonomy and purpose (Suryadi et al., 2024; Choi et al., 2012). Through this process, followers’ needs for autonomy and self-development are fulfilled, leading to enhanced individual competence and improved organizational performance (Shin, 2021; Woo and Moon, 2022). Ultimately, transformational leadership functions not only as a catalyst for achieving organizational objectives but also as a vital mechanism for fostering members’ long-term growth and sustaining a positive organizational culture.

In university sports teams, coaches occupy a uniquely complex leadership position, as they must pursue not only athletic excellence but also the educational objectives of fostering students’ academic achievement and character development. This dual mission renders leadership within university athletics inherently multifaceted and crucial. In such settings, coaches are expected to go beyond tactical game management to enact leadership that aligns athletic goals with the broader educational mission of the institution, while simultaneously promoting both individual growth and collective performance (Comeaux and Harrison, 2011).

Moreover, the university sports context differs markedly from typical organizational settings, featuring a flatter hierarchy, high interdependence among members, and a culture shaped by intangible, relational dynamics (Burton and Peachey, 2014). Within this unique environment, transformational leadership plays a pivotal role in enhancing team cohesion and performance by fostering shared goals and intrinsic motivation among athletes. However, despite its strong theoretical foundation and practical relevance, empirical research on transformational leadership in university athletics remains relatively limited. A systematic examination of how transformational leadership influences both team performance and individual development in university sports would provide meaningful theoretical insights, contributing to the refinement and extension of leadership theory within this distinct organizational context (Burton and Peachey, 2009).

To evaluate the effectiveness of transformational leadership within university sports teams, it is essential to examine its influence on group cohesion. A considerable body of research has demonstrated that transformational leadership effectively enhances team cohesion (Charbonneau et al., 2001; Kim and Cruz, 2016; Ryan, 2012). Group cohesion refers to the extent to which team members unite and collaborate toward shared objectives, serving as a critical determinant of both interpersonal dynamics and overall team performance (Carron and Chelladurai, 1981). Cohesion is generally conceptualized along two dimensions: social cohesion and task cohesion (Carron and Chelladurai, 1981). Social cohesion captures the affective bonds and interpersonal attraction among team members, emphasizing emotional connectedness, positive relationships, and a sense of belonging. In contrast, task cohesion pertains to the degree of collaboration and commitment members exhibit in pursuing collective performance goals, thereby acting as a key driver of strategic and competitive success (Prien, 2000).

In university sports, where teams strive to achieve both athletic excellence and academic success, task cohesion becomes particularly salient in optimizing team outcomes (Smith et al., 2013). However, prior studies have often conceptualized cohesion as a single, overarching construct rather than distinguishing between its social and task dimensions (Kao et al., 2019; Ryan, 2012). This lack of differentiation neglects the possibility that emotional bonding and cooperative behavior may stem from distinct psychological processes. Accordingly, the present study seeks to clarify the specific influence of transformational leadership on task cohesion within university sports teams, identifying the underlying mechanisms that facilitate this relationship.

However, the direct association between leadership and team outcomes may not sufficiently account for the intricate psychological processes involved. Theoretically, when potent mediating variables are introduced to explain these processes, the initial direct pathway may be weakened or rendered non-significant, suggesting that leadership influence is fully channeled through specific internal mechanisms (Baron and Kenny, 1986). Therefore, examining these intervening pathways is essential to move beyond a simplistic understanding of leadership effectiveness and to reveal how leaders fundamentally foster team collaboration.

According to transformational leadership theory, the effectiveness of leadership is fundamentally based on the establishment of trust between leaders and their followers (Bass, 1985). In particular, the idealized influence component manifests when a leader earns the trust and admiration of subordinates through moral integrity and exemplary behavior, thereby becoming a model for identification. This concept provides a theoretical foundation for explaining how transformational leadership directly influences followers’ trust (Bass and Avolio, 1994). Therefore, investigating the role of trust in the relationship between transformational leadership and team cohesion constitutes a theoretically grounded approach.

Trust functions as a central mechanism that activates the key elements of team cohesion, including expectations of the leader, mutual respect among members, open communication, and a cooperative climate. Team cohesion refers to the extent to which members are united and committed to achieving shared objectives, thereby fostering a sense of belonging and sustained mutual support within the team (Carron et al., 1998). For such interactional dynamics to develop, trust in the leader and among team members must first be established. Empirical evidence suggests that trust facilitates conflict resolution, information sharing, and responsible behavior within teams, all of which contribute to the reinforcement of cohesion (Dirks, 2000). Therefore, given that transformational leadership operates on a foundation of mutual trust, its influence on team cohesion can be understood as being mediated through trust.

A growing body of empirical evidence supports this mediating mechanism. Kao et al. (2019) identified a cross-level moderating effect, demonstrating that the impact of a coach’s transformational leadership on team cohesion varied according to the level of shared trust within the team. Kang and Oh (2015) further reported that trust in the leader positively influenced not only task cohesion but also athlete satisfaction. Likewise, Lim et al. (2010) showed that transformational leadership enhances both players’ trust and task cohesion. Extending beyond the context of sports, Lim and Kim (2014) found in the field of organizational behavior that transformational leadership strengthens interpersonal trust, which in turn mediates the relationship between leadership and team cohesion, ultimately facilitating knowledge sharing. Taken together, these findings indicate that the influence of transformational leadership on cohesion is generally realized through the mediating role of trust, suggesting that such a mediation model is not merely a methodological preference but a theoretically grounded framework.

Nevertheless, a key limitation of previous research lies in its predominant tendency to conceptualize trust as a unidimensional construct. In reality, trust within organizations is far more complex than simple emotional affinity; it is increasingly recognized that genuine trust develops only when it is anchored in perceptions of a leader’s competence and moral integrity. To address this conceptual gap, McAllister (1995) proposed a two-dimensional model of trust, distinguishing between cognitive and affective trust, and argued that the two dimensions exhibit distinct characteristics while maintaining a hierarchical relationship.

Cognitive trust is grounded in rational evaluations of a leader’s expertise, reliability, and consistency, serving as an initial condition that enables members to assess whether the leader is credible and dependable. In contrast, affective trust develops through ongoing interpersonal interactions and emotional bonds between leaders and followers, reflecting the emotional attachment that emerges over time. McAllister (1995) further proposed that cognitive trust precedes and facilitates affective trust, indicating that an initial, rational confidence in a leader can gradually evolve into deeper emotional commitment and cohesion within the team.

Unlike professional teams with established long-term contracts, university athletic teams operate within a highly condensed four-year cycle where athletes face intense pressure to secure professional career opportunities within a limited timeframe (Comeaux and Harrison, 2011). In this ‘performance-first’ environment, the formation of team task cohesion is likely driven more by a coach’s perceived competence and tactical reliability—cognitive trust—than by purely interpersonal emotional bonds—affective trust (Dirks, 2000; McAllister, 1995). As noted by Dirks (2000), the impact of trust in athletic settings is highly contingent upon situational demands and the collective focus on immediate outcomes. This suggests that in high-stakes competitive settings like university football, the functional necessity of expert leadership may render the impact of affective trust secondary or even non-significant when rational demands for competence are paramount.

Building on this theoretical framework, the present study extends prior research that has typically treated trust as a single mediating construct. Instead, it conceptualizes cognitive and affective trust as distinct yet interrelated dimensions and proposes a sequential mediation model in which transformational leadership first fosters cognitive trust, which subsequently gives rise to affective trust. This approach allows for a more nuanced examination of the psychological mechanisms through which transformational leadership influences task cohesion. By accounting for the structural complexity of trust, the study aims to advance a more integrative understanding of leadership effectiveness within university athletic teams.

### 1.1 Research hypotheses

*H1*: Perceived transformational leadership of university soccer coaches will positively predict team task cohesion.

*H2:* Cognitive trust will mediate the relationship between perceived transformational leadership and team task cohesion.

*H3*: Affective trust will mediate the relationship between perceived transformational leadership and team task cohesion.

*H4*: Cognitive trust and affective trust will sequentially mediate the relationship between perceived transformational leadership and team task cohesion.

## 2 Methods

### 2.1 Participants

The participants of this study were university soccer players affiliated with the Korea University Sports Federation (KUSF), a public organization under the Ministry of Culture, Sports and Tourism. These athletes are characterized by their serious professional commitment, as they compete in the national U-League with the primary goal of transitioning into professional soccer careers. University soccer teams represent a highly interdependent group context in which cooperation and interaction among members directly influence both athletic performance and overall team outcomes. While the current sample focuses on a specific demographic—male collegiate soccer players in South Korea—this deliberate focus allows for a rigorous examination of the structural relationships between leadership and team dynamics by minimizing potential confounding factors such as gender-specific leadership perceptions, cross-sport variations, and broad cultural differences. By establishing this contextual consistency, the study can more precisely isolate the psychological mechanisms through which transformational leadership fosters trust and task cohesion within a high-pressure, performance-oriented environment. Accordingly, university soccer teams provide an appropriate and empirically relevant context for examining the formation of team cohesion and its antecedent factors.

A non-probability purposive sampling method was employed. Specifically, active university soccer players affiliated with the Korea University Sports Federation (KUSF) who provided informed consent to participate were recruited. To enhance representativeness within this defined population, participants were drawn from various geographical regions and league divisions across South Korea. The online survey was conducted in March 2025, and after excluding incomplete or inattentive responses, a total of 362 valid questionnaires were retained for the final analysis.

The sample size (*N* = 362) was deemed sufficient for structural equation modeling (SEM), as it exceeds the recommended 10:1 to 15:1 ratio of participants to estimated parameters (Bentler and Chou, 1987). Furthermore, a post-hoc power consideration confirmed that the sample size provided sufficient statistical power (above 0.80) to yield reliable and stable findings for the hypothesized mediation paths, ensuring the robustness of the structural framework (Kline, 2023).

All study procedures were reviewed and approved by the Institutional Review Board (IRB) of Dongguk University (Approval No. DUIRB-2025-01-12) in compliance with ethical research standards. Participation was entirely voluntary. Prior to data collection, participants were fully informed about the purpose and procedures of the study and were required to submit an online consent form before participation. The survey was conducted anonymously, and no personally identifiable information was collected, ensuring the confidentiality and privacy of all participants.

### 2.2 Measurements

This study employed validated and reliable measurement instruments to empirically examine the relationships among transformational leadership, trust (cognitive and affective trust), and team task cohesion within a university soccer organization. All items were rated on a five-point Likert scale, ranging from 1 (“strongly disagree”) to 5 (“strongly agree”).

#### 2.2.1 Transformational leadership

Transformational leadership was measured using items from the Multifactor Leadership Questionnaire (MLQ-5X) developed by Bass and Avolio (1990) and adapted into Korean by Jeon (2013). To ensure the scale’s applicability to the collegiate athletic context, specific contextual adaptations were made to the items. For example, general organizational terms were replaced with ‘My Leader’, and technical elaborations were added to clarify abstract concepts within a sports framework. A notable adaptation is seen in the intellectual stimulation subscale (Item 9), where ‘important standards’ was expanded to include ‘training methods, team management, and match strategies’ to better resonate with athletes’ daily experiences. The internal consistency of the transformational leadership scale in this study was Cronbach’s *α* = 0.956, indicating excellent reliability.

#### 2.2.2 Trust

In this study, trust was conceptualized and measured based on McAllister’s (1995) two-dimensional model, distinguishing between cognitive trust and affective trust. Cognitive trust refers to the degree of confidence in a leader’s competence and expertise, whereas affective trust reflects the extent to which the leader is perceived to understand and care for the emotional needs of team members. The measurement items were adapted from McAllister (1995), Tsai et al. (2012), and Jeon (2013), comprising six items for cognitive trust and five items for affective trust. To ensure the scale’s applicability to the university sports environment, the items were specifically contextualized by the researcher; for instance, general organizational terms (e.g., ‘supervisor’ or ‘person’) were substituted with ‘my coach’ to reflect the coach-athlete relationship. Furthermore, the phrasing of several items was slightly modified to better suit the athletic context, such as referring to ‘professional skills’ as ‘coaching and performance. The internal consistency reliability (Cronbach’s *α*) in this study was 0.948 for cognitive trust and 0.904 for affective trust, indicating excellent reliability for both constructs.

#### 2.2.3 Team task cohesion

Team task cohesion was measured using the Group Environment Questionnaire (GEQ) originally developed by Carron et al. (1985), which was subsequently adapted and refined by Lee (1992) to better reflect the specific characteristics and cultural context of Korean sports teams. To ensure the instrument’s applicability to university soccer, the items were specifically worded to capture the task-oriented collaboration and strategic alignment inherent in a competitive team environment. For instance, the scale includes items such as ‘Our team members are fully aware of the team’s match strategies’ and ‘The team members collectively analyze the game content after it ends.’ The final instrument consisted of 15 items, and the internal consistency reliability (Cronbach’s alpha) obtained in this study was 0.940, indicating a high level of reliability.

### 2.3 Statistical analysis

#### 2.3.1 Preliminary analysis

In the preliminary stage, descriptive statistical analyses were conducted to calculate the mean, standard deviation, skewness, and kurtosis of all study variables in order to assess the normality of the data distribution. To identify potential issues of multicollinearity, multiple regression analyses were performed, to calculate the Variance Inflation Factor (VIF) and Tolerance indices. Following the conservative criteria suggested by Hair et al. (2010), VIF values below 5.0 and Tolerance values above 0.2 were used as thresholds to ensure the absence of significant multicollinearity issues. Additionally, correlation analyses were used to explore the bivariate relationships among variables.

Subsequently, a measurement model analysis was conducted to evaluate the model’s overall goodness of fit. The model fit was assessed using several established indices, including the Chi-square (χ^2^) statistic, Comparative Fit Index (CFI), Tucker–Lewis Index (TLI), and Root Mean Square Error of Approximation (RMSEA). Following the conventional criteria suggested by Hu and Bentler (1999), model fit was considered acceptable when CFI and TLI exceeded 0.90 and RMSEA was below 0.08.

The standardized factor loadings for each indicator were examined to verify measurement validity, with factor loadings of 0.50 or higher indicating satisfactory item reliability (Hair et al., 2010). Based on these analyses, the construct validity, convergent validity, and discriminant validity of the measurement instruments were assessed and confirmed.

#### 2.3.2 Mediation model analysis

To test the dual mediation effects of trust in the relationship between transformational leadership and team task cohesion, structural equation modeling (SEM) was employed. The statistical significance of the indirect effects was evaluated using the phantom variable approach, and a bootstrapping procedure with 5,000 resamples was conducted. The bias-corrected 95% confidence interval (CI) was used to determine the significance of the mediation effects; mediation was considered significant when zero was not contained within the lower and upper limits (LLCI, ULCI) of the confidence interval.

Through this analysis, the study examined how athletes’ perceptions of their coaches’ transformational leadership influence team task cohesion, and elucidated the mediating roles of cognitive and affective trust in this process.

## 3 Results

### 3.1 Descriptive statistics

The participants consisted of 362 male university soccer players. Their distribution across competitive levels was relatively balanced, with 54.4% (*n* = 197) competing in U-League 1 and 45.6% (*n* = 165) in U-League 2, representing a broad spectrum of the collegiate competitive environment. In terms of academic year, first-year players were the most prevalent (57.2%), followed by second-year (26.0%), third-year (12.2%), and fourth-year players (4.7%). Regarding team roles, the sample included starting players (29.0%), rotation players (34.5%), and substitutes (36.5%), ensuring that diverse perspectives on leadership and team dynamics were captured across different levels of field involvement.

The results of the descriptive statistical analysis indicated that the absolute values of skewness ranged from 0.303 to 0.526, while the absolute values of kurtosis ranged from 0.067 to 0.570 (Table 1). According to the criteria suggested by Kline (2023), data are considered to meet the assumption of normality when the absolute value of skewness is less than 3 and that of kurtosis is less than 10. Therefore, all variables measured in this study were deemed to satisfy the assumption of normality.

**Table 1:** Descriptive statistics.

Variables	Mean	*SD*	Skewness	Kurtosis
Transformational leadership	3.996	0.727	−0.526	−0.141
Cognitive trust	4.013	0.825	−0.494	−0.498
Affective trust	3.828	0.849	−0.522	−0.067
Team task cohesion	3.924	0.686	−0.303	−0.570

### 3.2 Correlation analysis

Before testing the hypotheses, a correlation analysis was conducted to examine the relationships among the major study variables. The results are presented in Table 2. All variables were found to have statistically significant positive correlations with one another.

**Table 2:** Correlation matrix.

Variables	1	2	3	4
1. Transformational leadership	1			
2. Cognitive trust	0.858**	1		
3. Affective trust	0.775**	0.802**	1	
4. Team task cohesion	0.614**	0.653**	0.587**	1

**p < 0.01.

Specifically, transformational leadership showed strong positive correlations with both cognitive trust (*r* = 0.858, *p* < 0.001) and affective trust (*r* = 0.775, *p* < 0.001), suggesting that athletes’ perceptions of their coaches’ transformational leadership are closely linked to both cognitive and emotional trust in the leader. Additionally, cognitive and affective trust were also highly correlated (*r* = 0.802, *p* < 0.001), indicating that rational confidence in a leader’s competence and consistency is closely associated with the formation of emotional bonds with that leader. However, because some correlations exceeded 0.80, we conducted a rigorous multicollinearity diagnosis by calculating the Variance Inflation Factor (VIF) and Tolerance indices. The results showed that all VIF values were between 3.044 and 4.615, which are well within the conservative threshold of 5.0 (Hair et al., 2010), and Tolerance values were above 0.2. These findings confirm that multicollinearity did not pose a significant threat to the structural model estimation despite the high bivariate correlations. Finally, team task cohesion was significantly and positively correlated with transformational leadership (*r* = 0.614, *p* < 0.001), cognitive trust (*r* = 0.653, *p* < 0.001), and affective trust (*r* = 0.587, *p* < 0.001). These findings indicate that athletes’ perceptions of transformational leadership and trust in their coaches are strongly related to team members’ cohesion toward achieving shared performance goals.

### 3.3 Measurement model analysis

#### 3.3.1 Model fit

A Confirmatory Factor Analysis (CFA) was conducted using AMOS 21.0 to examine whether the measurement items appropriately represented their underlying constructs. The four-factor model—comprising transformational leadership, cognitive trust, affective trust, and team task cohesion—demonstrated an acceptable model fit: χ^2^ = 393.255, df = 162, CFI = 0.967, TLI = 0.962, RMSEA = 0.063, SRMR = 0.033.

All indices met or exceeded recommended cutoff values, indicating that the measurement model adequately explained the observed data and that the construct validity of the study’s measurement scales was established.

#### 3.3.2 Convergent validity

To assess convergent validity, the Average Variance Extracted (AVE) and Composite Reliability (CR) for each latent construct were calculated (Table 3). According to Fornell and Larcker (1981), convergent validity is supported when AVE values exceed 0.50 and CR values exceed 0.70.

**Table 3:** Results of convergent validity analysis.

Variables	Item	Estimate	*SE*	*CR*	Factor loading	*AVE*	*CR*
Transformational leadership	Idealized influence	1.000			0.804	0.747	0.922
	Inspirational motivation	1.111	0.059	18.937***	0.846		
	Intellectual stimulation	1.251	0.062	20.138***	0.882		
	Individualized consideration	1.273	0.059	21.477***	0.921		
Cognitive trust	Cognitive Q1	1.000			0.854	0.753	0.948
	Cognitive Q2	1.023	0.051	20.140***	0.823		
	Cognitive Q3	1.110	0.050	22.055***	0.865		
	Cognitive Q4	1.180	0.050	23.497***	0.894		
	Cognitive Q5	1.201	0.051	23.663***	0.898		
	Cognitive Q6	1.086	0.049	22.281***	0.870		
Affective trust	Affective Q7	1.000			0.794	0.665	0.908
	Affective Q8	1.044	0.051	20.378***	0.911		
	Affective Q9	0.897	0.063	14.162***	0.692		
	Affective Q10	0.876	0.047	18.575***	0.852		
	Affective Q11	0.924	0.053	17.425***	0.813		
Team task cohesion	Cohesion 1	1.000			0.874	0.717	0.927
	Cohesion 2	0.989	0.052	19.153***	0.812		
	Cohesion 3	1.029	0.049	20.872***	0.851		
	Cohesion 4	1.013	0.049	20.648***	0.847		
	Cohesion 5	0.947	0.046	20.757***	0.848		

***p < 0.001.

The results indicated that all standardized factor loadings were above 0.50, reflecting satisfactory item reliability. All constructs demonstrated AVE values of at least 0.665 and CR values of at least 0.908, thus exceeding the recommended thresholds. These results confirm that the measurement indicators converged appropriately on their respective latent constructs, providing strong evidence of convergent validity for all instruments used in this study.

#### 3.3.3 Discriminant validity

Discriminant validity was assessed following the criteria proposed by Fornell and Larcker (1981), which require that the Average Variance Extracted (AVE) for each construct exceed the squared correlation coefficients between that construct and all others. In other words, the shared variance between distinct constructs must be smaller than the amount of variance explained by each construct’s own indicators, thereby demonstrating that the latent variables are empirically distinct.

As shown in Table 4, the square roots of the AVE values for all constructs were greater than the corresponding inter-construct correlations, confirming that each latent variable was statistically distinguishable from the others. Hence, the measures used in this study satisfied the criterion for discriminant validity.

**Table 4:** Results of discriminant validity analysis.

Variables	1	2	3	4
1. Transformational leadership	**0.747**			
2. Cognitive trust	0.736	**0.753**		
3. Affective trust	0.601	0.643	**0.665**	
4. Team task cohesion	0.378	0.426	0.345	**0.717**

The bold values along the diagonal represent the Average Variance Extracted (AVE) for each construct. The values below the diagonal indicate the squared correlations between the constructs.

### 3.4 Verification of the research model

This study examined the effect of transformational leadership on team task cohesion and tested the mediating and serial mediating effects of cognitive trust and affective trust in this relationship. To this end, path analysis was conducted using Structural Equation Modeling (SEM) with AMOS 21.0. The significance of the mediating effects was evaluated through bootstrapping with 5,000 resamples, and 95% confidence intervals were estimated (Preacher et al., 2007). An indirect effect was considered statistically significant when zero was not included in the confidence interval.

Additionally, the phantom variable technique was employed to analyze individual mediation effects. This approach allowed for a more precise estimation of bootstrapped confidence intervals for specific mediation paths and enabled independent verification of each indirect effect within complex models containing multiple mediators (Macho and Ledermann, 2011).

The model fit indices demonstrated that the research model had a satisfactory level of fit: χ^2^ (162) = 393.225, CFI = 0.967, TLI = 0.962, and RMSEA = 0.063, confirming that the model was statistically acceptable.

#### 3.4.1 Path analysis

**Figure 1:**
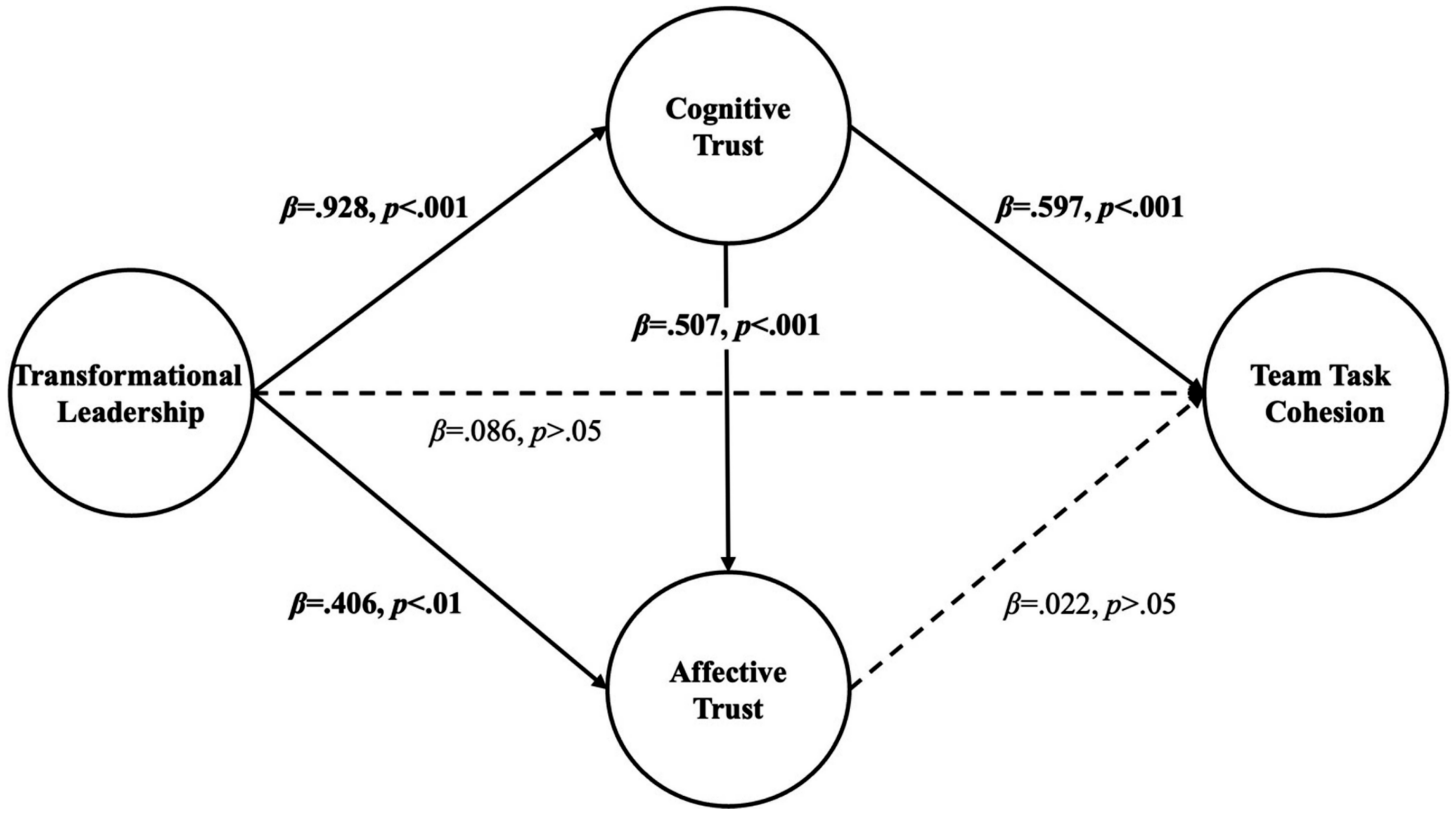
Multiple mediation model illustrating the pathways from transformational leadership to team task cohesion via cognitive trust and affective trust. Solid lines indicate significant effects (*p* < 0.01 or *p* < 0.001), while the dashed lines represent 0non-significant paths (*p* > 0.05). All path coefficients are presented as standardized values.

The research model and path coefficients are illustrated in Figure 1, with detailed results of the path analysis presented in Table 5. First, transformational leadership had a significant positive effect on cognitive trust (B = 0.888, *β* = 0.928, *p* < 0.001). This finding indicates that leader behaviors such as articulating a vision, providing individualized consideration, and offering intellectual stimulation enhance members’ positive perceptions of others’ competence, expertise, and reliability.

**Table 5:** Results of path analysis.

Path		*B*	*S. E.*	*C. R.*	*β*
Transformational Leadership	→ Cognitive trust	0.888	0.041	21.471	0.928***
	→ Affective trust	0.416	0.112	3.72	0.406***
	→ Team task cohesion	0.075	0.140	0.534	0.086 (n.s.)
Cognitive trust	→ Affective trust	0.544	0.118	4.621	0.507***
	→ Team task cohesion	0.539	0.150	3.6	0.597***
Affective trust	→ Team task cohesion	0.019	0.099	0.189	0.022 (n.s.)

***p < 0.001; n.s. = non-significant.

Next, transformational leadership also exerted a significant positive effect on affective trust (B = 0.416, *β* = 0.406, *p* < 0.001), suggesting that leadership behaviors contribute to fostering emotional bonds, empathy, and a sense of belonging among team members. However, the direct effect of transformational leadership on team task cohesion was not statistically significant (B = 0.075, *β* = 0.086, *p* = 0.594). This result implies that the influence of leadership on team cohesion may operate primarily through indirect pathways, such as the mediating role of trust, rather than through direct effects.

Furthermore, cognitive trust had a significant positive effect on affective trust (B = 0.544, *β* = 0.507, *p* < 0.001), indicating that when team members have confidence in others’ competence and judgment, their emotional closeness and sense of camaraderie are also likely to be strengthened. In addition, cognitive trust showed a significant positive effect on team task cohesion (B = 0.539, *β* = 0.597, *p* < 0.001), suggesting that trust in team members’ expertise promotes collaboration and commitment to shared tasks.

In contrast, affective trust did not have a significant effect on team task cohesion (B = 0.019, *β* = 0.022, *p* = 0.850). This suggests that emotional intimacy and interpersonal affection alone may be insufficient to directly enhance task-related cohesion or cooperative engagement within the team.

#### 3.4.2 Mediating effect

The results of the mediation analysis examining the indirect effects of cognitive trust and affective trust in the relationship between transformational leadership and team task cohesion are presented in Table 6.

**Table 6:** Results of mediating effect analysis.

Path	*B*	*S. E.*	95% Confidence interval	
			Lower limit	Upper limit
Transformational Leadership → Cognitive Trust → Team Task Cohesion	0.479	0.157	0.193	0.816
Transformational Leadership → Affective Trust → Team Task Cohesion	0.008	0.049	−0.075	0.124
Transformational Leadership → Cognitive Trust → Affective Trust → Team Task Cohesion	0.009	0.055	−0.123	0.103

First, the indirect effect of transformational leadership on team task cohesion through cognitive trust was significant (B = 0.479, 95% CI = [0.193, 0.816]). This indicates that transformational leadership enhances members’ cognitive trust—confidence in the leader’s competence and reliability—which, in turn, strengthens team task cohesion.

Second, the indirect effect through affective trust alone was not significant (B = 0.008, 95% CI = [−0.075, 0.124]), as the confidence interval included zero. This suggests that the pathway in which transformational leadership influences team task cohesion solely through affective trust does not yield a statistically meaningful effect.

Third, the sequential mediation effect through both cognitive trust and affective trust was also nonsignificant (B = 0.009, 95% CI = [−0.123, 0.103]), as zero was included in the confidence interval. This finding implies that the indirect pathway in which transformational leadership enhances cognitive trust, subsequently increases affective trust, and ultimately leads to higher team task cohesion did not operate at a statistically significant level. Despite this insignificant sequential result, it is noteworthy that the specific path from cognitive trust to affective trust exhibited a robust effect size (*β* = 0.507), as detailed in the path analysis. This underscores that while the overall serial link to task cohesion was not established, cognitive trust remains a critical and powerful prerequisite for the development of affective trust among athletes.

Taken together, these results indicate that only the indirect pathway through cognitive trust alone was statistically significant. Neither affective trust by itself nor the sequential mediation of cognitive and affective trust demonstrated a significant mediating effect in the relationship between transformational leadership and team task cohesion.

## 4 Discussion

This study examined how university soccer players’ perceptions of their coaches’ transformational leadership influence team task cohesion, with particular attention to the mediating roles of cognitive and affective trust. The findings revealed that the direct effect of transformational leadership on team task cohesion was not statistically significant. Instead, cognitive trust emerged as a key mediating factor, whereas affective trust did not exert a significant direct influence on task cohesion. The sequential mediation of cognitive and affective trust was only partially supported. In this section, these results are interpreted in light of previous research, and their theoretical and practical implications are discussed in depth, particularly with respect to the contextual characteristics of university sports and the multidimensional mechanisms underlying leadership effectiveness.

The first hypothesis predicted that coaches’ transformational leadership would have a direct positive effect on the task cohesion of university soccer teams. However, the structural equation modeling (SEM) analysis revealed that this direct path was not statistically significant. This finding suggests that while transformational leaders articulate a clear vision and provide individualized consideration (Bass, 1985; Bass and Riggio, 2006), these behaviors do not automatically or immediately translate into task-oriented collaboration. Instead, the influence of leadership is fully channeled through specific internal psychological mechanisms, demonstrating a complete mediation effect (Baron and Kenny, 1986). In this model, transformational leadership serves as a distal antecedent that sets the stage for team functioning, but its functional impact on task cohesion is contingent upon the formation of trust.

This discrepancy with prior research such as studies by Kao et al. (2019) and Choi et al. (2011), which reported direct positive influences of leadership on cohesion can be interpreted through the unique structural and psychological context of university athletics. First, university soccer teams operate within a dual-commitment environment where athletes must balance high-level athletic performance with academic and personal development (Jeong and Kim, 2017). In this multifaceted setting, the immediate impact of leadership may be diffused by players’ pursuit of diverse goals, making the establishment of deeper psychological foundations—specifically trust—a prerequisite for aligning individual needs with collective objectives (Prien, 2000). Second, because university sports are characterized by high interdependence and a flat hierarchy (Burton and Peachey, 2014), leadership effectiveness is validated through followers’ internalizing the coach’s vision rather than formal authority (Choi et al., 2012). These results imply that transformational leadership must be mediated by the cognitive evaluation of a leader’s competence to effectively drive task-oriented teamwork (Smith et al., 2013).

The second hypothesis proposed that cognitive trust would mediate the relationship between transformational leadership and team task cohesion, and both sub-paths (transformational leadership → cognitive trust and cognitive trust → task cohesion) were found to be statistically significant. This indicates that transformational leadership fosters players’ rational confidence in their coach’s competence, expertise, and behavioral consistency, and that such cognitive trust, in turn, enhances commitment and collaboration toward team tasks. This mediating pathway remained the most robust within the structural framework, corroborating the “competence-first” demand of elite sports (Dirks, 2000). In the high-stakes environment of university athletics, where players face intense pressure to secure professional opportunities within a limited timeframe (Comeaux and Harrison, 2011), athletes tend to engage more deeply when they perceive their coach as strategically and tactically dependable. This finding reinforces McAllister’s (1995) proposition that professional expertise and reliability are essential antecedents of trust in performance-driven organizations.

The third hypothesis proposed that affective trust would mediate the relationship between transformational leadership and team task cohesion. However, the path from affective trust to task cohesion was not statistically significant, leading to a rejection of this hypothesis. In contrast, the sub-path from transformational leadership to affective trust was significant, suggesting that while transformational leadership enhances emotional bonds and relational closeness, such affective trust does not directly translate into stronger task cohesion. This divergence from some previous studies (e.g., Ahn and Kim, 2009) highlights a critical boundary condition: while affective trust fosters emotional comfort and satisfaction, it may be secondary to the functional requirements of goal-oriented role execution (Prien, 2000). Particularly in competitive settings, coaches who overemphasize “emotional care” while neglecting to demonstrate professional excellence may fail to enhance team combat effectiveness. These findings imply that affective trust supports team morale as a complementary process, whereas cognitive trust remains the principal driver of strategic success.

The fourth hypothesis proposed that cognitive trust and affective trust would sequentially mediate the relationship between transformational leadership and team task cohesion. This pathway was partially supported, as cognitive trust served as a significant precursor to affective trust, consistent with McAllister’s (1995) hierarchical model. However, the overall dual mediation failed to reach significance because the final link from affective trust to task cohesion remained weak. This indicates that while rational confidence may eventually evolve into emotional commitment, this progression does not necessarily translate into superior functional coordination in the short-term, result-oriented context of university soccer. Consequently, coaches should adopt a stage-based leadership approach: first establishing a foundation of cognitive trust through professional consistency and tactical expertise to ensure task-oriented unity, and then cultivating affective trust to sustain long-term psychological well-being (Dirks, 2000; McAllister, 1995).

### 4.1 Suggestions for future research

This study has several limitations that should be acknowledged, and based on these, directions for future research are proposed.

First, the sample consisted exclusively of male university soccer players in Korea, creating a high degree of sample homogeneity. While this allowed for a controlled analysis of the collegiate context, it limits the generalizability of the findings to other genders, age groups, or sports. Future studies should include female athletes, professional teams, and various team sports to account for diverse psychological and environmental variables.

Second, the cross-sectional design restricts the ability to infer causal relationships. Subsequent studies should employ longitudinal designs to trace how perceptions of leadership evolve and how the accumulation of trust influences the development of team cohesion over time within dynamic competitive environments.

Third, although this study intentionally focused on task cohesion to investigate goal-oriented collaboration, the adapted GEQ is capable of assessing both task and social dimensions. Given that affective trust is likely to play a more salient role in shaping social cohesion, team satisfaction, and emotional commitment, future research should incorporate both dimensions to provide a more holistic understanding of team dynamics. Furthermore, designing alternative structural models centered on affective outcomes or using moderation and multi-group analyses could further elucidate how the role of trust varies across contexts.

Fourth, despite satisfying the variance inflation factor (VIF) criteria, the relatively high correlations between transformational leadership and cognitive trust (*r* = 0.858) and between cognitive and affective trust (*r* = 0.802) suggest possible conceptual overlap. To address this, future research should refine measurement instruments to ensure clear conceptual delineation and employ more rigorous verification methods, such as comparing the hypothesized model with alternative measurement models (e.g., merging highly correlated factors) through confirmatory factor analysis (CFA).

Beyond methodological improvements, this study carries important practical implications. At the coaching level, cognitive trust can be enhanced through clear tactical visions and transparent communication, while affective trust should be cultivated through psychological support and individualized consultations. At the athlete level, programs that strengthen shared leadership and sub-leadership groups are recommended to facilitate internal trust formation. Integrating quantitative analyses with qualitative approaches, such as in-depth interviews, would yield a deeper understanding of how trust is sustained, ultimately contributing to a trust-based leadership model applicable across various sports levels.

## 5 Conclusion

This study aimed to examine the impact of transformational leadership on team task cohesion within the competitive organizational context of university soccer teams and to elucidate the mediating effects of cognitive and affective trust. By distinguishing trust into two psychological dimensions—rational evaluation (cognitive trust) and emotional attachment (affective trust)—this research highlighted that the influence of leadership extends beyond direct behavioral effects and operates through the psychological mechanisms of followers.

The analysis revealed that transformational leadership did not exert a direct effect on task cohesion; rather, only the indirect pathway through cognitive trust was statistically significant. In contrast, affective trust was found to be influenced by transformational leadership either directly or indirectly via cognitive trust, yet neither the direct path to task cohesion nor the dual mediation effect was significant. Moreover, cognitive trust had a significant positive effect on affective trust, confirming the hierarchical and interdependent nature of the two trust dimensions.

These findings broaden the interpretive scope of sports leadership theory and provide practical insights into effective coaching behaviors. Specifically, the results indicate that transformational leadership enhances team functioning not merely through vision and motivation, but by enabling athletes to perceive their coach as a competent and dependable leader. This perception, in turn, fosters collaborative task engagement and reinforces cohesion. Accordingly, transformational leadership can be understood as a trust-building mechanism that strengthens both cognitive confidence and emotional connection within the team.

From a practical perspective, the findings suggest that coaches seeking to enhance team cohesion should first focus on building cognitive trust rather than solely providing emotional support. Consistent training methods, clear performance standards, and fair leadership practices allow athletes to view their coach as capable and dependable, thereby increasing cooperation and indirectly fostering affective bonds among team members. In sum, trust-based leadership serves as a critical bridge linking leadership behaviors to sustained team cohesion in university sports contexts.

## Data Availability

The raw data supporting the conclusions of this article will be made available by the authors, without undue reservation.
